# Maize *opaque* mutants are no longer so opaque

**DOI:** 10.1007/s00497-018-0344-3

**Published:** 2018-07-05

**Authors:** Shanshan Zhang, Junpeng Zhan, Ramin Yadegari

**Affiliations:** School of Plant Sciences, University of Arizona, Tucson, AZ 85721 USA

**Keywords:** Maize, Endosperm, Opaque, Seed storage proteins, Gene regulatory network

## Abstract

The endosperm of angiosperms is a zygotic seed organ that stores nutrient reserves to support embryogenesis and seed germination. Cereal endosperm is also a major source of human calories and an industrial feedstock. Maize *opaque endosperm* mutants commonly exhibit opaque, floury kernels, along with other abnormal seed and/or non-seed phenotypes. The opaque endosperm phenotype is sometimes accompanied by a soft kernel texture and increased nutritional quality, including a higher lysine content, which are valuable agronomic traits that have drawn attention of maize breeders. Recently, an increasing number of genes that underlie *opaque* mutants have been cloned, and their characterization has begun to shed light on the molecular basis of the opaque endosperm phenotype. These mutants are categorized by disruption of genes encoding zein or non-zein proteins localized to protein bodies, enzymes involved in endosperm metabolic processes, or transcriptional regulatory proteins associated with endosperm storage programs.

## Introduction

Endosperm is a product of double fertilization in the female gametophyte (embryo sac), and it functions as a nutritive organ to support embryogenesis and seedling development (Olsen and Becraft 2013; Olsen 2004). In cereals, the endosperm comprises the largest portion of the seed, and is a major source of food, feed, and industrial raw materials (Becraft and Gutierrez-Marcos 2012; Lopes and Larkins 1993). In maize, the endosperm initially differentiates into four main cell types, which are termed starchy endosperm (SE), aleurone (AL), embryo-surrounding region (ESR), and basal endosperm transfer layer (BETL) (Becraft and Gutierrez-Marcos 2012; Leroux et al. 2014; Zhan et al. 2015, 2017). Each cell type has unique morphological and functional properties. For example, the SE, as the central and largest portion of the endosperm, accumulates starch and storage proteins; AL is the epidermal cell layer that synthesizes hydrolases to mobilize the starch and storage proteins to support seedling establishment during germination; and the BETL mediates transport of nutrients into the kernel (Becraft and Gutierrez-Marcos 2012; Gontarek and Becraft 2017; Zhan et al. 2017). The proper development of these cell types is critical to the overall function of the endosperm and development of the seed.

In typical dent maize varieties, the mature SE has two distinct areas, a peripheral, vitreous region, and a central, starchy region (Gibbon and Larkins 2005). The latter has poorer light transmittance than the former. If the vitreous region is unable to form during endosperm maturation, it generates an opaque or floury endosperm phenotype (Gibbon and Larkins 2005; Larkins et al. 2017). Over the past century, a variety of maize mutants with an opaque or floury endosperm phenotype have been identified and are termed “opaque” [e.g., *opaque1*–*17* (*o1*, *o2*, *o5*–*o7*, *o9*–*o11*, *o13*–*o17*), recessive], “floury” [e.g., *floury1*–*4* (*fl1*–*fl4*), semidominant], or other assorted names [e.g., *defective endosperm* (*De*)-*B30* and *Mucronate* (*Mc*), dominant; *mutator*-*tagged opaque 140* (*mto140*), recessive] (Gibbon and Larkins 2005; Larkins et al. 2017). The diversity of these mutations raises the question as to what forms the basis of the opaque endosperm phenotype (referred to as opaque phenotype hereafter). The opaque phenotype is commonly associated with altered protein bodies (PBs) and/or starch granules—that is, mutants with an opaque phenotype display defective PB structure or function to varying degrees, depending on the causal gene. As an example, many opaque-type mutants (collectively referred to as opaque mutants hereafter) are defective in the expression or accumulation of prolamins which are the most abundant seed storage protein in maize (> 60%) (Wu and Messing 2014). Prolamins, called zeins in maize, are encoded by single and multiple gene families to produce four distinct classes of proteins: α-, β-, γ-, and δ-zeins (Coleman and Larkins 1999; Larkins et al. 2017). Recently, a number of genes that underlie the well-known *opaque* mutants have been characterized (Table 1). These mutants provide novel insights into the molecular mechanisms associated with the opaque kernel phenotype and the larger question of how endosperm development is regulated. The nature of the underlying genes generally falls into three main categories: (1) zein coding sequences (e.g., *FL2*, *FL4*, *De*-*B30*, and *Mc*) and genes encoding non-zein proteins associated with PBs (e.g., *O1*, *O10*, and *FL1*); (2) genes encoding enzymes involved in endosperm metabolic processes (e.g., *O5*, *O6*, *O7*, and *MTO140*); and (3) transcriptional regulatory genes (e.g., *FL3*, *O2*, and *O11*; Table 1).

**Table 1:** Summary of maize traditional *opaque endosperm* mutants mapped

Mutant	Locus	Gene name^a^	Protein type	References
o1*	Zm00001d052110	–	Myosin XI motor	Wang et al. (2012)
o2*	Zm00001d018971	–	bZIP TF	Schmidt et al. (1987, 1990)
o5*	Zm00001d020537	*MGD1*	Monogalactosyldiacylglycerol synthase	Myers et al. (2011)
o6*	Zm00001d010056	*PRO1*	D1-pyrroline-5-carboxylate synthetase	Wang et al. (2014b)
o7*	Zm00001d026649	*AAE3*	Acyl-CoA synthetase	Miclaus et al. (2011) and Wang et al. (2011)
o10*	Zm00001d033654	–	Cereal-specific non-zein PB protein	Yao et al. (2016)
o11*	Zm00001d003677	–	bHLH TF	Feng et al. (2018)
fl1**	Zm00001d003398	–	Non-zein PB protein	Holding et al. (2007)
fl2**	Zm00001d049243	–	22-kD α-zein	Coleman et al. (1997) and Gillikin et al. (1997)
fl3**	Zm00001d009292	–	PLATZ TF	Li et al. (2017)
fl4**	Zm00001d048851	–	19-kD α-zeins	Wang et al. (2014a)
*De*-B30***	Zm00001d019158	–	19-kD α-zeins	Kim et al. (2004)
Mc***	Zm00001d005793	–	16-kD γ-zein	Kim et al. (2006)
mto140*	Zm00001d014734	*AroDH*-*1*	Arogenate dehydrogenase	Holding et al. (2010)

*De*-*B30* defective endosperm-B30, *fl* floury, *Mc* Mucronate, *o* opaque, *PB* protein body

Inheritance of mutant: *recessive; **semidominant; ***dominant

^a^Only the gene names that are different from the corresponding mutant names are shown

## The opaque endosperm mutant phenotype is often due to changes in storage protein synthesis, deposition, and metabolism

Mutations in some storage-protein genes, or genes associated with PB development and metabolism can produce an opaque endosperm. Mutations in zein genes produce either a dominant or semidominant opaque or floury phenotype. Three mutants, including *fl2*, *fl4,* and *De*-*B30*, were shown to result from point mutations in the signal peptide cleavage site of a 22-kD α-zein (Coleman et al. 1997; Gillikin et al. 1997) and two 19-kD α-zein proteins (Kim et al. 2004; Wang et al. 2014a). These mutations alter zein protein deposition and generate irregularly shaped PBs (Coleman et al. 1997; Gillikin et al. 1997; Kim et al. 2004; Lending and Larkins 1992; Wang et al. 2014a). In contrast, the *Mc* mutant results from a 38-bp deletion in a 16-kD γ-zein gene, creating a frame shift in the coding sequence that gives rise to misshapen PBs (Kim et al. 2006). Knockdown of one or a combination of zein genes using RNA interference (RNAi) can also generate an opaque phenotype (Guo et al. 2013; Larkins et al. 2017; Segal et al. 2003). Therefore, the dominant or semidominant opaque phenotypes are primarily associated with zein gene loss of function that is critical for proper PB formation.

Mutations in some genes, including *O1*, *O10,* and *FL1*, can result in an opaque phenotype via alterations to proper PB assembly. *O1* encodes a myosin XI motor-like protein (Wang et al. 2012); although zein protein synthesis is not detectably affected, the *o1* mutant exhibits PBs that are smaller and somewhat misshapen compared to wild type (Wang et al. 2012). A role in proper formation of PBs, particularly in the ring-shaped distribution of 22-kD α-zeins and the 16-kD γ-zein, has also been shown for O10 (Yao et al. 2016). The single *o10* mutant allele described thus far, which encodes a cereal-specific PB protein, has a point mutation (a G-to-A transition) at the 3′ end of intron 6 (Yao et al. 2016). This mutation produces a truncated O10 protein, due to retention of intron 6 and premature termination of its synthesis (Yao et al. 2016). The truncated protein is able to interact with a subset of α-zeins and γ-zeins through its N-terminal amino acid residues, as its wild-type counterpart, but it lacks the ability to localize to the ER and become deposited in PBs because it lacks the requisite C-terminal transmembrane domain (Yao et al. 2016). *FL1* is also linked with PBs and zeins. It encodes a membrane protein that resides in the ER surrounding the PBs, and likely facilitates localization of 22-kD α-zeins in PBs (Holding et al. 2007).

Opaque phenotypes also have resulted from mutations in genes encoding enzymes involved in metabolic processes that can be linked with zein protein accumulation and/or PB development. The *o5* mutant is defective in a Monogalactosyldiacylglycerol synthase (MGD1), which is required for normal amyloplast and chloroplast functions and normal morphology of starch granules (Myers et al. 2011). *O6* encodes a D1-pyrroline-5-carboxylate synthetase that catalyzes synthesis of proline from glutamic acid (Wang et al. 2014b). The *o6* [also named *proline responding 1* (*pro1*)] mutant has been reported to block biogenesis of proline, resulting in a general reduction in protein synthesis, an inhibition of cell proliferation, and an associated down-regulation of cyclin gene expression (Wang et al. 2014b). As expected, the level of zein (all of which are high in proline) protein synthesis is dramatically reduced in *o6* (Wang et al. 2014b). The *o7* mutant, which has been mapped to the *AAE3* gene that encodes an acyl-activating enzyme-like protein, shows a preferential reduction of 19-kD α-zeins due to an unknown mechanism (Miclaus et al. 2011; Wang et al. 2011). Finally, the *mto140* mutant, defective in the arogenate dehydrogenase 1 (AroDH-1) involved in tyrosine synthesis, has been shown to affect accumulation of all families of zeins (Holding et al. 2010). The nature of this group of genes further supports a link between dysregulated zein gene expression and PB formation, with the consequent generation of an opaque endosperm phenotype.

## Mutations in regulatory genes associated with endosperm storage programs

**Fig. 1:**
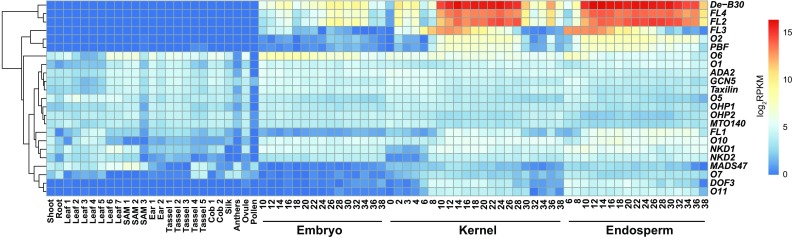
Spatial and temporal expression patterns of *opaque* and related genes in maize vegetative and reproductive structures. Relative levels of gene mRNAs visualized using a heat map hierarchically clustered on Euclidean distance. The normalized RNA-Seq reads (in reads per kilobase per million mapped reads, RPKM) for selected tissues/organs from a published expression atlas of maize inbred B73 including shoot, root, leaf, shoot apical meristem (SAM), ear, tassel, cob, silk, anther, ovule, pollen, whole kernels, endosperm, and embryos of different developmental stages (in DAP) (Chen et al. 2014), were used

**Fig. 2:**
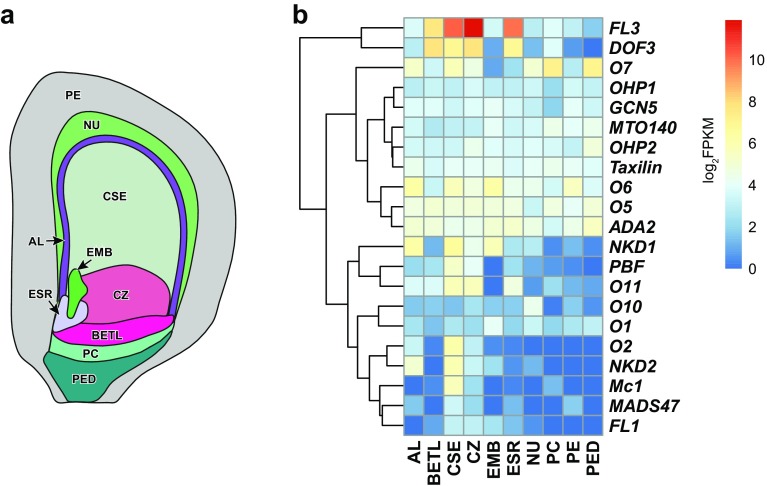
Spatial expression pattern of *opaque* and related genes in maize kernel. **a** Graphic representation of an 8-DAP maize kernel showing the compartments used in **b** from Zhan et al. (2015). **b** Relative levels of gene mRNAs visualized using a heat map hierarchically clustered on Euclidean distance. The normalized RNA-Seq reads (in fragments per kilobase of transcript per million mapped reads, FPKM) of 8-DAP kernel compartments, including the aleurone (AL), the embryo-surrounding region (ESR), the basal endosperm transfer layer (BETL), two subregions of the starchy endosperm [SE; central starchy endosperm (CSE) and conducting zone (CZ)], embryo (EMB), nucellus (NU), placento-chalazal region (PC), pericarp (PE), and the vascular region of the pedicel (PED) (Zhan et al. 2015), were used

The best characterized transcription factor (TF) gene whose loss-of-function mutants can produce an opaque phenotype is *Opaque*-*2* (*O2*). *O2* is specifically expressed in the endosperm as early as 6 days after pollination and encodes a bZIP-family TF (Fig. 1) (Li et al. 2014; Schmidt et al. 1990). Previous studies showed that O2 directly regulates many target genes associated with storage functions, including zeins, through binding to a number of conserved *cis*-motifs collectively known as the O2 box (Cord Neto et al. 1995; Frizzi et al. 2010; Hartings et al. 2011; Hunter et al. 2002; Jia et al. 2007, 2013; Li et al. 2015; Muth et al. 1996; Schmidt et al. 1987, 1990, 1992; Zhang et al. 2015, 2016). Thus far, a number of protein partners of O2 have been identified that include both annotated TFs [e.g., the PROLAMIN-BOX BINDING FACTOR (PBF), the O2-heterodimerizing proteins (OHP1 and OHP2), MADS47, ALTERATION/DEFICIENCY IN ACTIVATION2 (ADA2)] and non-TF proteins [e.g., Taxilin and GENERAL CONTROL OF NITROGEN5 (GCN5)] (Bhat et al. 2004; Hwang et al. 2004; Jin et al. 2014; Pysh et al. 1993; Pysh and Schmidt 1996; Qiao et al. 2016; Vicente-Carbajosa et al. 1997; Wang et al. 1998; Yilmaz et al. 2009; Zhang et al. 2012). Except for a few genes that are primarily expressed in the endosperm (e.g., *PBF* and *O2* itself), most of these proteins are encoded by genes that are ubiquitously expressed throughout the plant life cycle (Fig. 1). Within the endosperm, these genes show diverse spatial patterns of expression (Fig. 2). These data indicate that the gene expression programs associated with an opaque phenotype are regulated by TFs programmed for specialized roles in the whole endosperm or even in individual compartments (cell types) of the endosperm, and also TFs that may have broader roles in regulation of transcription in different developmental contexts.

PBF is a DOF-family TF protein that binds the prolamin box (P box) and co-regulates a subset of target genes with O2 (Hwang et al. 2004; Vicente-Carbajosa et al. 1997; Wang et al. 1998; Zhang et al. 2015, 2016). The O2 paralogs, OHP1 and OHP2 can form heterodimers with O2, and have been shown to co-activate zein genes with O2 in a partially redundant manner (Zhang et al. 2015). However, O2 is considered as the major regulator of α-zeins, while OHPs are key regulators of the 27-kD γ-zein gene (Zhang et al. 2015). In agreement with their roles as regulators of storage-protein gene expression, RNAi lines of PBF and OHPs (*PbfRNAi* and *OhpRNAi*, respectively) show a reduction of zein synthesis, alleviation of the opaque phenotype, and display additive or synergistic defects in combination with *o2* mutants (Zhang et al. 2015, 2016). Different from PBF and OHPs, MADS47 is unable to activate zein expression on its own, but can synergistically activate zein gene transcription with O2 (Qiao et al. 2016). RNAi lines of MADS47 show a reduction of zein synthesis and a decreased size of PBs (Qiao et al. 2016). In contrast, Taxilin has been shown to interact with O2 to modulate the transcriptional regulatory role of the O2 protein through changing its subcellular distribution (Zhang et al. 2012). O2, GCN5 (a putative histone acetyltranferase), and ADA2 (a putative transcriptional adaptor protein) have been reported to interact with one another and co-regulate expression of target genes (Bhat et al. 2003, 2004).

The *nkd* mutants, which were initially identified based on defects in AL development, also show an opaque phenotype (Gontarek et al. 2016; Yi et al. 2015). *NKD1* and *NKD2* are recently duplicated genes that encode INDETERMINATE-domain-family TFs that can directly activate a number of genes that were also shown to be regulated by O2, including a 22-kD α-zein gene (Gontarek et al. 2016). NKD1 and NKD2 also can directly activate *O2* itself (Gontarek et al. 2016). Furthermore, *NKD1* expression can be directly activated by DOF3 (Qi et al. 2017). Interestingly, both the *nkd* mutants and RNAi lines of *DOF3* (*Dof3RNAi*) exhibit defects in SE and AL cell differentiation (Gontarek et al. 2016; Qi et al. 2017; Yi et al. 2015). *NKD* genes, *DOF3*, *PBF*, and *O2*, have recently been identified as direct targets of O11, which is a bHLH family TF encoded by a gene expressed specifically in the endosperm (Fig. 1) (Feng et al. 2018). Moreover, O2 and O11 have been shown to antagonistically regulate a number of common target genes including *CYTOSOLIC PYRUVATE ORTHOPHOSPHATE DIKINASE1* (*cyPPDK1*) and *cyPPDK2* (Feng et al. 2018). In addition to an opaque phenotype, the *o11* mutant also manifests an abnormal interface between the endosperm and embryo, which is consistent with detection of several ESR-specific genes (e.g., *YODA*, encoding a MAPKK kinase) as direct target genes of O11 (Feng et al. 2018). These observations suggest that cellular differentiation in AL, ESR, and internal SE cells could be coordinately regulated through an O11-DOF3-NKD1/2-O2-PBF regulatory axis.

The nature of genes regulated by O2 suggests that the O2-regulated gene network plays important roles in controlling kernel nutritional quality and yield (Zhang et al. 2016). Recent profiling of mutants and knockdown lines of O2 and its nuclear partners (*o2*, *PbfRNAi*, and *OhpRNAi*) showed O2-network genes exhibit diverse spatial and temporal patterns of expression and functionalities (Frizzi et al. 2010; Hartings et al. 2011; Hunter et al. 2002; Jia et al. 2007, 2013; Li et al. 2015; Zhang et al. 2016). As mentioned above, a key subset of target genes includes the zein multi-gene family. Mutations in or down-regulation of some zein genes can improve the lysine deficiency of wild-type maize, and thereby increase its nutritional quality (Frizzi et al. 2010; Hunter et al. 2002). The *LKR/SDH* gene encoding a lysine-ketoglutarate reductase/saccharopine dehydrogenase is activated by O2 (Kemper et al. 1999). This enzyme functions in the lysine degradation pathway during late endosperm development (Kemper et al. 1999). Endosperm-specific knockdown of *LKR/SDH* using RNAi resulted in up to 20-fold increase in free lysine content (Houmard et al. 2007). Therefore, the increased lysine content of *o2* mutants can be partially explained by the down-regulation of the *LKR/SDH* gene. With respect to the potential role of O2 in regulating starch and protein content, a number of genes encoding enzymes in the starch synthesis pathway have been shown to be either directly [e.g., *STARCH SYNTHASEIII* (*SSIII*)] or indirectly [e.g., *SSIIa* and *STARCH*-*BRANCHING ENZYME1* (*SBE1*)] activated by O2 (Zhang et al. 2016). In addition, O2 can also transcriptionally activate *b*-*32*, which encodes an RNA N-glycosidase that likely functions as a defense-related protein by inhibiting protein synthesis through its ribosome inactivating activity (Bass et al. 1992; Lohmer et al. 1991). The role of *b*-*32* in the context of a regulatory program primarily associated with storage protein and starch accumulation remains elusive. A number of other questions remain to be addressed with respect to the full scope of O2’s role as a regulator of these diverse functionalities, including a detailed view of the associated gene regulatory networks and the full repertoire of its molecular partners. Interestingly, a recent analysis of the *fl3* mutant identified a PLATZ-family TF as a regulator of a subset of functionalities that overlap with O2 (Li et al. 2015, 2017). *FL3* is preferentially expressed in SE cells based on mRNA localization and regulates many tRNAs, 5S rRNAs, and other genes involved in translation, ribosome assembly and function, the unfolded protein response (UPR), and nutrient reservoir activity (e.g., zein genes and starch biosynthetic pathway genes) (Li et al. 2017). The regulatory function of FL3 likely occurs through its interaction with components of transcriptional machinery, including transcription factor class C 1 (TFC1) and RNA polymerase III subunit 53 (RPC53), two critical factors of the RNA polymerase III (RNAPIII) complex (Li et al. 2017). Interestingly, *fl3* exhibits a semidominant phenotype, which is likely due to its parent-of-origin-dependent expression pattern, with the maternal allele being expressed and the paternal allele silenced specifically in the endosperm (Li et al. 2017).

As part of the effort to breed for maize varieties with increased lysine content but a harder endosperm (in contrast to normal *o2* mutants which have starchy and soft endosperm and therefore are more susceptible to damage by fungi or insects), a number of genetic suppressors of *o2* (*o2* modifiers) have been discovered that enabled development of “quality protein maize (QPM),” which manifests a high lysine content and vitreous endosperm (Gibbon and Larkins 2005; Larkins et al. 2017). Genetic markers linked to *o2* modifiers have been identified, including the 27-kD γ-zein, which has been suggested to play an essential role in modification of the *o2* phenotype (Holding et al. 2011; Liu et al. 2016; Yuan et al. 2014). However, the underlying molecular mechanisms are yet to be fully elucidated.

## Future perspectives

Analysis of maize opaque mutants indicates a tight association between a starchy endosperm phenotype of the kernel and altered storage protein deposition, that is, an altered size, number, and/or structure of endosperm PBs. Several recently published transcriptome analyses of *opaque* mutants revealed that many of them display altered accumulation of other storage products or dysregulated expression of genes associated with their synthesis and/or metabolism. For example, carbohydrate and lipid metabolism is perturbed in *o2*, *o7*, and *o11* mutants (Feng et al. 2018; Frizzi et al. 2010; Hartings et al. 2011; Jia et al. 2007, 2013; Li et al. 2015; Wang et al. 2011). Therefore, the opaque phenotype is often associated with perturbation in primary metabolism. UPR is another common feature of the dysregulated genes in opaque mutants. Generally, UPR is a homeostatic response to alleviate ER stress due to interference with protein folding, or as a result of adverse environmental conditions (Howell 2013). Recently, UPR has been shown to have a higher activity in the central endosperm (corresponding to the starchy region in mature endosperm) as compared with peripheral endosperm (corresponding to the vitreous region in mature endosperm) (Gayral et al. 2017). Interestingly, opaque mutants, defective in forming vitreous endosperm, also show upregulation of genes involved in UPR. These include mutants with defective zein genes (e.g., *fl2*, *fl4*, *De*-*B30*, and *Mc*), transcriptional regulators (e.g., *o2* and *fl3*), and others (e.g., *o1* and *o5*) (Gibbon and Larkins 2005; Hunter et al. 2002; Li et al. 2017). Together, these observations suggest a mechanistic connection between the opaque phenotype and processes associated with storage product metabolism and the UPR.

In addition to seed storage-function-associated biological processes (discussed above), many opaque mutants also show defects in developmental processes of both seed and non-seed tissues. First, the coincident perturbation of SE/AL/ESR differentiation with storage compound accumulation in the *nkd*, *Dof3RNAi,* and *o11* mutants suggest coordinated regulation of these developmental processes, which may occur temporally in a partially overlapping manner. Second, in addition to their respective mutant phenotypes described above, the *nkd* mutants show pleiotropic seed phenotypes, including a multilayered, partially differentiated AL and occasional vivipary (Gontarek and Becraft 2017; Gontarek et al. 2016; Yi et al. 2015). Moreover, the *o11* mutant exhibits abnormal embryo (scutellum) morphology (Feng et al. 2018). These observations suggest that the opaque phenotype is also linked with other key seed developmental programs, such as mitotic proliferation of endosperm cells, embryogenesis, and seed maturation. However, whether these developmental processes are also dysregulated in other opaque mutants is unclear. Third, in contrast to other opaque mutants, which generally do not display vegetative defects, the *o5* mutant seedlings display a pale green or albino phenotype, the *mto140* mutant shows slightly retarded vegetative growth, while *o6* shows a reduction in seedling height and root length that can be rescued by application of l-proline (Holding et al. 2010; Myers et al. 2011; Wang et al. 2014b). Therefore, detailed analyses of mutant phenotypes in seed and non-seed tissues, including the morphology and cytology of differentiating/differentiated endosperm cell types and the associated gene expression programs in comparison with wild type, will be needed to fully understand the relationship between the opaque phenotype and its cellular and molecular basis.

Recent studies of TFs (e.g., O2, O11, NKDs, and FL3) with corresponding mutants that show an opaque phenotype led to identification of additional regulatory proteins that function upstream, downstream, or as partners with the TFs. Some of these proteins are implicated in regulation of storage programs and/or other key programs of seed development or the endosperm’s response to abiotic stress. For example, NKDs regulate the *Viviparous*-*1* (*VP1*) gene that encodes an ABI3-VP1 TF family required for proper seed maturation and germination (Gontarek et al. 2016; Hoecker et al. 1995); O2 and O11 directly regulate the bZIP-family *G*-*BOX BINDING FACTOR1* (*GBF1*) gene that is involved in response to hypoxia (Feng et al. 2018; Li et al. 2015; Vetten and Ferl 1995). In addition, nuclear proteins GCN5, ADA2, and MADS47 interact with O2 to co-regulate downstream gene expression (Bhat et al. 2003, 2004; Qiao et al. 2016). Moreover, although the molecular mechanisms associated with *o2* modification are still unclear, the *o2* modifiers constitute potentially useful tools for understanding the genetic processes underlying the opaque phenotype. Further understanding of the molecular basis of the opaque phenotype will likely require an understanding of the function of additional regulatory hubs, particularly TF proteins, their respective networks, and other genetic factors (and their respective gene networks) that can modify the opaque, starchy endosperm phenotype.
